# Intradiscal injection of monosodium iodoacetate induces intervertebral disc degeneration in an experimental rabbit model

**DOI:** 10.1186/s13075-021-02686-6

**Published:** 2021-12-08

**Authors:** Takao Sudo, Koji Akeda, Koki Kawaguchi, Takahiro Hasegawa, Junichi Yamada, Nozomu Inoue, Koichi Masuda, Akihiro Sudo

**Affiliations:** 1 Department of Orthopaedic Surgery, Mie University Graduate School of Medicine, 2-174 Edobashi, Tsu City, Mie 514-8507 Japan; 2 Department of Orthopedic Surgery, Rush Medical College, Chicago, IL 60612-3833 USA; 3 Department of Orthopaedic Surgery, University of California, San Diego, 9500 Gilman Dr, La Jolla, 92093-0863 USA

**Keywords:** Intervertebral disc, Disc degeneration, Monosodium iodoacetate, μCT, MRI, Histology, Rabbit, Animal model

## Abstract

**Background:**

Establishing an optimal animal model for intervertebral disc (IVD) degeneration is essential for developing new IVD therapies. The intra-articular injection of monosodium iodoacetate (MIA), which is commonly used in animal models of osteoarthritis, induces cartilage degeneration and progressive arthritis in a dose- and time-dependent manner. The purpose of this study was to determine the effect of MIA injections into rabbit IVDs on the progression of IVD degeneration evaluated by radiographic, micro-computerized tomography (micro-CT), magnetic resonance imaging (MRI), and histological analyses.

**Methods:**

In total, 24 New Zealand White (NZW) rabbits were used in this study. Under general anesthesia, lumbar discs from L1–L2 to L4–L5 had a posterolateral percutaneous injection of MIA in contrast agent (CA) (L1–L2: CA only; L2–L3: MIA 0.01 mg; L3–L4: 0.1 mg; L4–L5: 1.0 mg; L5–L6: non-injection (NI) control). Disc height was radiographically monitored biweekly until 12 weeks after injection. Six rabbits were sacrificed at 2, 4, 8, and 12 weeks post-injection and processed for micro-CT, MRI (T2-mapping), and histological analyses. Three-dimensional (3D) disc height in five anatomical zones was evaluated by 3D reconstruction of micro-CT data.

**Results:**

Disc height of MIA-injected discs (L2–L3 to L4–L5) gradually decreased time-dependently (*P* < 0.0001). The disc height of MIA 0.01 mg-injected discs was significantly higher than those of MIA 0.1 and 1.0 mg-injected discs (*P* < 0.01, respectively). 3D micro-CT analysis showed the dose- and time-dependent decrease of 3D disc height of MIA-injected discs predominantly in the posterior annulus fibrosus (AF) zone. MRI T2 values of MIA 0.1 and 1.0 mg-injected discs were significantly decreased compared to those of CA and/or NI controls (*P* < 0.05). Histological analyses showed progressive time- and dose-degenerative changes in the discs injected with MIA (*P* < 0.01). MIA induced cell death in the rabbit nucleus pulposus with a high percentage, while the percentage of cell clones was low.

**Conclusions:**

The results of this study showed, for the first time, that the intradiscal injection of MIA induced degenerative changes of rabbit IVDs in a time- and dose-dependent manner. This study suggests that MIA injection into rabbit IVDs could be used as an animal model of IVD degeneration for developing future treatments.

## Background

Low back pain (LBP), a prevalent worldwide health problem afflicting patients ranging from children to the elderly, is a major cause of disability [1]. Clinically, intervertebral disc (IVD) degeneration is an important cause of pain in LBP patients [2, 3]. The pathophysiology of human IVD degeneration is complex and multifactorial, and environmental and endogenous factors under genetic predisposition are considered to initiate the degenerative changes of human IVDs (see review in [4]).

It is important to establish an optimal animal model for IVD degeneration that mimics the biological process of human IVD degeneration in order to elucidate the pathological mechanisms of degenerative disc diseases and to develop novel treatments for them.

The IVD consists of the gelatinous nucleus pulposus (NP) surrounded by the annulus fibrosus (AF), a concentrically organized lamella structure of collagen fibers. The NP is rich in an extracellular matrix (ECM) that mainly contains the hydrophilic proteoglycan (PG) “aggrecan” and type II collagen [5]. The cells in human IVDs undergo a phenotypic change in the NP from notochordal cells to chondrocyte-like cells in childhood [6]. With increasing age or degeneration, there is an increase in cell proliferation (formation of cell clusters) and an increase in cell death [7]. These cellular changes are accompanied by biochemical changes in the ECM constituents, including a decrease in water and PG content that leads to tissue fibrosis in the NP [4].

Consequently, the number and extent of intervertebral clefts and tears are increased [8]. With the recent progress of image analysis technology, these biochemical and histological features of human IVD degeneration are now able to be evaluated by magnetic resonance imaging (MRI) [9, 10] and computed tomography (CT) images [11]. These cellular and biochemical changes also influence disc function and structure characterized by disc height narrowing [12, 13]. Numerous animal models of IVD degeneration have been reported and have been compared regarding the histological, biochemical, and structural characteristics of human IVD degeneration [14].

Monosodium iodoacetate (MIA) is commonly used in animal models of osteoarthritis (OA) (see review in [15]). MIA inhibits glyceraldehyde-3-phosphate dehydrogenase activity in chondrocytes, resulting in disruption of glycolysis and eventual cell death [16] and apoptosis [17]. The intra-articular injection of MIA induces a reduction in the number of chondrocytes and morphological and histological changes of articular cartilage, similar to those changes in human osteoarthritis (OA) [18, 19]. Udo et al. recently reported that MIA injection into rat knee joints induced cartilage degeneration and progressive arthritis in a dose- and time-dependent manner [20]. The injection of a low amount of MIA induced cartilage degeneration without bone destruction, as found in the pathology of an early stage of OA. By contrast, a high dose of MIA showed a severe degradation of cartilage with the destruction of subchondral bone [20].

IVDs and articular cartilage share remarkably similar anatomical composition, biochemical features, and molecular processes of matrix degeneration. Suh et al. [21] recently investigated the effect of MIA injection into rat IVDs, mainly on behavior analysis during 6 weeks post-injection, and showed that the intradiscal injection of MIA (4.0 mg) affected pain behavior with severe degenerative changes of rat IVDs. Rapid and severe degeneration with complete cell loss is not optimum to study the effect of biologics. In addition, a large animal model, such as rabbits, is suitable to control doses of materials to be injected without backflow from a needle hole after injection. It is essential to retain some functional cells to investigate cell-mediated tissue regeneration in a preclinical model.

Therefore, we hypothesized that (i) the intradiscal injection of MIA induces the degeneration of rabbit IVDs in a dose- and time-dependent manner, and (ii) the proper dose of MIA can be used in an animal model to study IVD degeneration for preclinical studies of IVD therapy.

The purpose of this study was to determine the effect of MIA injection into rabbit IVDs on the progression of IVD degeneration evaluated by radiographic, micro-computed tomography (micro-CT), magnetic resonance imaging (MRI), and histological analyses, and to establish a new rabbit IVD degeneration model.

## Methods

### Procedure

**Fig. 1:**
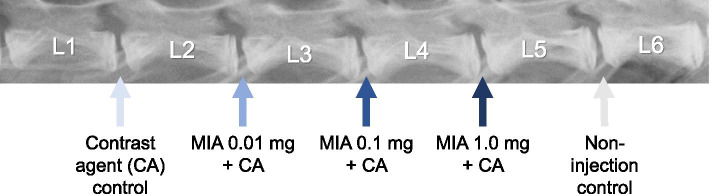
Schema of monosodium iodoacetate (MIA) injection into rabbit intervertebral discs (IVDs). The contrast agent (CA) or MIA in CA was injected into rabbit IVDs. Each disc had a posterolateral percutaneous injection of CA or MIA in 10 μl of CA with a 31G needle using a micro syringe. L5–L6 had no operation and was used as a non-injection (NI) control

This study was conducted in strict accordance with recommendations in the Guide for the Care and Use of Laboratory Animals of the National Institutes of Health. The protocol of this study was approved by the Institutional Animal Care and Use Committee. Twenty-four New Zealand white rabbits (female), ranging from 2.9 to 3.4 kg in body weight (SLC, Hamamatsu, Japan), were used in this study. Rabbits were housed in separate cages under standard conditions with a light-dark cycle (12 h–12 h) and dry-bulb room temperature at 22–24° and provided ad libitum access to tap water and food pellets daily. Rabbits were anesthetized by an intramuscular injection of ketamine hydrochloride (25 mg/kg; Ketalar®; Daiichi Sankyo, Tokyo, Japan) mixed with xylazine (5 mg/kg; Selactar®, Bayer, Tokyo, Japan). Lateral plain radiographs were obtained to determine baseline IVD height values before the treatment. Under single fluoroscopy (C-arm image intensifier, Philips Med System), rabbits were then placed into a lateral prone position, and a nonionic, isotonic contrast agent (CA) (Iotrolan 240, Bayer Global, Leverkusen, Germany) or MIA (305-53-3, Sigma-Aldrich, St. Louis, MO, USA) was injected into the rabbit IVDs from the posterolateral to the center percutaneously using micro syringes with a 31G needle (Ito Corporation, Fuji, Japan). L1–L2 discs received 10 μl of CA only, L2–L3 discs received MIA 0.01 mg (in 10 μl CA), L3–L4 MIA 0.1 mg (in 10 μl CA), and L4–L5 MIA 1.0 mg (in 10 μl CA). L5–L6 discs were used as non-injection (NI) controls (Fig. 1). The use of the lumbar spine lowest levels (L6–L7) was avoided to eliminate possible influences of the lumbosacral junction.

Six rabbits were euthanized at 2, 4, 8, and 12 weeks post-injection, and the spinal columns (L1 to L6 vertebra) were harvested. At each time point, three spinal columns were processed for micro-computed tomography (CT) and another three for magnetic resonance imaging (MRI), followed by histological analysis.

### Radiographic analysis of disc height

**Fig. 2:**
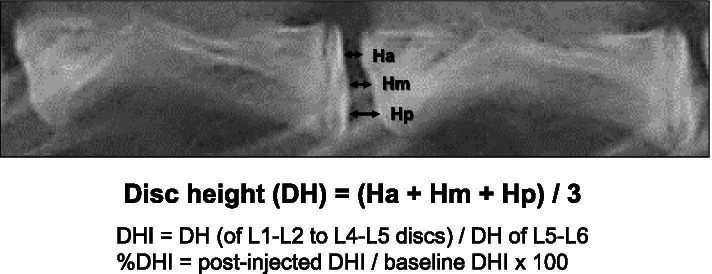
Radiographic measurement of disc height. Anterior disc height (Ha), middle disc height (Hm), and posterior disc height (Hp) were measured and calibrated. Disc height (DH) was calculated as (Ha +Hm +Hp) /3. The disc height index (DHI) was defined as: DH (L1–L2, L2–L3, L3–L4, or L4–L5 disc) / DH of L5–L6 disc (non-injection (NI) control). Change in DHI was expressed as percentage DHI (%DHI) and normalized to the measured baseline DHI: % DHI = (post-injection DHI/baseline DHI) × 100

To assess the height of each disc, lateral radiographs of the lumbar segment were taken just before injection and at 2-week intervals after that until the animals were euthanized. Under general anesthesia, a lateral lumbar radiograph was taken using fluoroscopy. A 10-mm metal wire was placed at the height of the rabbit spine in the lateral recumbent position as a marker for calibration. All radiographic images were independently analyzed using an image analysis software, ImageJ (US National Institutes of Health, Bethesda, MD, USA), by an orthopedic researcher blinded to the treatment groups. The IVD height was measured using the method of Masuda et al. [22], with a modification. Anterior disc height (Ha), middle disc height (Hm), and posterior disc height (Hp) were measured and calibrated (Fig. 2). Disc height (DH) was calculated as follows: (Ha+Hm+Hp) / 3 (Fig. 2). The disc height index (DHI) was defined as: DH (of L1–L2, L2–L3, L3–L4, or L4–L5 discs) / DH of L5–L6 disc (NI control). Change in DHI was expressed as percentage DHI (%DHI) and normalized to the measured baseline DHI: % DHI = (post-injection DHI/baseline DHI) × 100.

### Micro-CT analysis

**Fig. 3:**
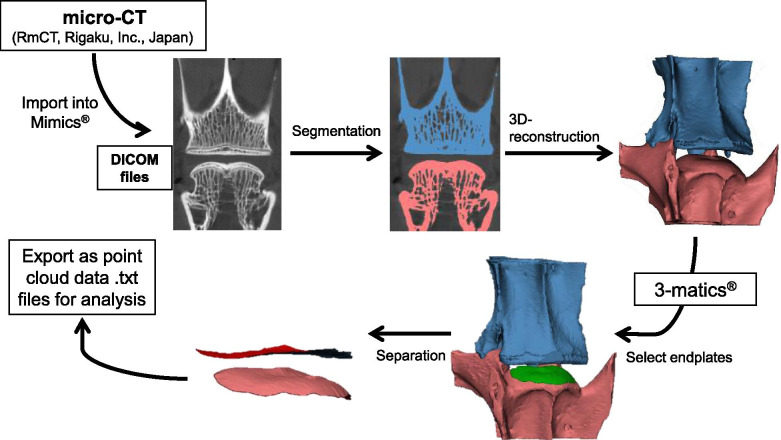
Creation of a three-dimensional computed tomography (3D CT) reconstruction model of the rabbit lumbar spine

Lumbar spinal columns with surrounding soft tissues harvested at each time point (*n* = 3) were CT-scanned in an X-ray micro-CT scanner (Rigaku, RmCT, Tokyo, Japan). The X-ray source operates at 75 kV and 100 μA, scan time 17 s, and voxel size 24 μm. Digital Imaging and Communications in Medicine (DICOM) data were reconstructed to three-dimensional (3D) images of vertebral body-disc-vertebral body units in Mimics 17.0 (Mimics, Materialise Inc., Leuven, Belgium). Then, isolation of the endplates was completed in 3-matics (Materialise Inc.), and endplates were exported as point cloud data (Fig. 3). For measuring the 3D-disc height (3D-DH), the least distances from one point in the point cloud model of the inferior endplate to the points in the superior endplate were calculated using a custom-written Visual C++ program [23, 24]. The average of least distance was calculated by repeating this procedure for all points in the inferior endplate, and then the averaged distance was defined as the 3D-DH of the whole disc. Next, the IVD was separated into five anatomical areas (posterior, left-lateral [L-lateral], right-lateral [R-lateral], anterior, and nucleus pulposus [NP]) that represent the AF and NP footprints ideally. The 3D-DH of each area was measured as described above [23, 24].

In order to evaluate the differences of 3D-DH among the five zones, the percentage of 3D-DH of all five zones against those of L5–L6 discs were calculated as 3D-DHI: (3D-DH of each disc) / (3D-DH of L5–L6) × 100 (%). 3D-DHI was evaluated in all the discs injected with CA or MIA after 2, 4, 8, and 12 weeks post-injection. All five zones were divided into four groups depending on the degree of 3D-DHI as follows: 3D-DHI<70%, 70%≦3D-DHI<80%, 80%≦3D-DHI<90%, 90%≦3D-DHI.

### MRI analysis

L1 to L6 vertebrae with surrounding soft tissues were isolated and wrapped by plastic cling film to prevent dehydration and kept in an ice box until subjected to quantitative transverse relaxation time (T2) MRI analysis as previously reported [25]. MRI was performed using a 3.0-Tesla imager (Achieva 3.0T; Philips, Amsterdam, The Netherlands) with a 3-inch birdcage extremity coil (Philips). The room temperature was kept at 24 °C during the MRI scan. Quantitative T2 mapping was performed by using a multi-echo spin-echo sequence in the sagittal plane. Scanning parameters were the following: time-to-repeat (TR) = 2500 ms; time-to-echo (TE) = 20, 40, 400 ms (20 TEs) ms; field of view = 10 cm; slice thickness = 3 mm; image matrix = 560 × 560; and number of excitations = 1. The total scanning time per sample was 9 min 22 s. For creating color-coded T2 maps, MRI images at multiple TE were imported into the medical image processing software (EV Insite, PSP corporation, Tokyo, Japan) with a T2 mapping plug-in. Mean signal intensities were determined in the regions of interest (ROI) framing the outer border of the AF on T2-weighted images taken at TE of 100 ms [25].

### Histological analysis

Following micro-CT and MRI assessment, the experimental IVDs were excised from the vertebral body-disc-vertebral body unit, and each IVD was fixed in 4% paraformaldehyde for 14 days at 4 °C and then permeated in HCL-based decalcified reagent (K-CX, Falma, Inc., Tokyo, Japan), embedded in paraffin, sectioned, and assessed by conventional histology. Mid-sagittal sections (5 μm) of each IVD were stained with hematoxylin and eosin, and safranin-O and fast green. Blinded to the experiment, an observer analyzed the histological sections and graded them using the recently established standardized histopathology scoring system of rabbit IVD degeneration [26–28].

### Cell counting of “survived” cells in the rabbit nucleus pulposus

Cells in the NP area with clear hematoxylin-stained nuclei within the eosin-stained cytoplasm were defined as “survived” cells (living cells) [29]. Cells with nuclei with an abnormal appearance were defined as “dead cells.” The number of “survived” cells was quantified by manually counting from three fields of view in the NP area (anterior, center, and posterior) of microscopic images per sample at ×200 magnification.

### Statistical analysis

Differences in %DHI, 3D-DHI, and MRI T2-values were assessed for statistical significance by two-way repeated-measures analysis of variance (ANOVA) followed by the Bonferroni or Tukey’s Honest Significant Difference (HSD) post hoc test. Histological grading scores were assessed by the Kruskal-Wallis test for inter-group comparisons and the Friedman test for temporal changes. All data are expressed as mean ± standard deviation (SD). All the statistical analyses were performed using IBM Statistical Package for Social Sciences Software (SPSS) Statistics (IBM Japan, Tokyo). The accepted level of significance was *P* < 0.05.

## Results

### Change in radiographic disc height

**Fig. 4:**
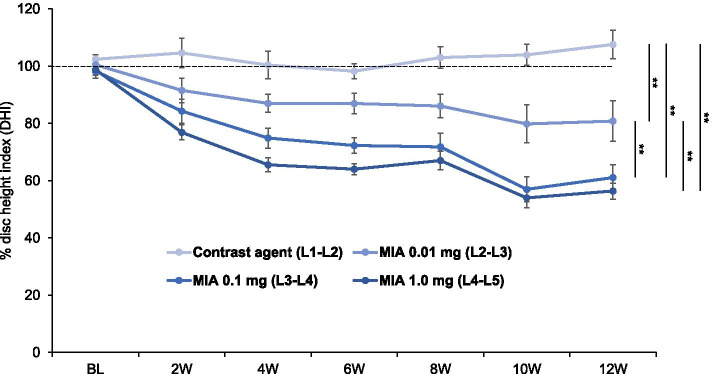
Change in percent disc height index (%DHI). Disc height (DH) was measured every 2 weeks by radiography in rabbit discs injected with the designated dose of monosodium iodoacetate (MIA) in contrast agent (CA) (L2–L3 MIA 0.01 mg, L3–L4 MIA 0.1 mg, and L4–L5 MIA 1.0 mg) and converted to percentage of disc height (%DHI). The mean %DHI of the discs decreased time-dependently (*P* < 0.01) and was significantly lower compared to that of the CA control (L1–L2) (*P* < 0.01, respectively). Data are expressed as mean ± standard error of the mean (SEM). Two-way analysis of variance and post hoc Bonferroni multiple comparisons test were used to compare all groups. **P < 0.01

The mean %DHI of discs injected with MIA (L2–L3, L3–L4, and L4–L5) decreased time-dependently (*P* < 0.01) and was significantly lower compared to that of the CA control (L1–L2) (*P* < 0.01, respectively) (Fig. 4). The %DHI of MIA 0.01 mg-injected discs (L2–L3) was significantly higher than that of MIA 0.1 mg-injected discs (L3–L4) and MIA 1.0 mg-injected discs (L4–L5) (*P* < 0.01, respectively); however, no significant difference was identified between MIA 0.1 mg-injected discs (L3–L4) and MIA 1.0 mg-injected discs (L4–L5) (Fig. 4).

The intradiscal injection of MIA 0.01 mg induced a significant decrease of %DHI to approximately 87% of baseline at 4 weeks post-injection; this continued to decrease to approximately 81% at 12 weeks post-injection. The %DHI of MIA 0.1 mg- and 1.0 mg-injected discs significantly decreased to approximately 75% and 66% of baseline at 4 weeks post-injection, and approximately 61% and 56% at 12 weeks post-injection. The timepoint analysis showed that %DHI of MIA 0.01 mg-injected discs was significantly higher than that of MIA 0.1 mg-injected discs at 6 weeks (*P* < 0.01) and 10 weeks (*P* < 0.05) post-injection and MIA 1.0 mg-injected discs at 4 weeks (*P* < 0.01), 6 weeks (*P* < 0.01), 8 weeks (*P* < 0.05), 10 weeks (*P* < 0.01), and 12 weeks (*P* < 0.05) post-injection. On the other hand, there were no significant differences in %DHI between the discs injected with MIA 0.1 mg and 1.0 mg from baseline to 12 weeks post-injection.

### Change in three-dimensional (3D) disc height

**Fig. 5:**
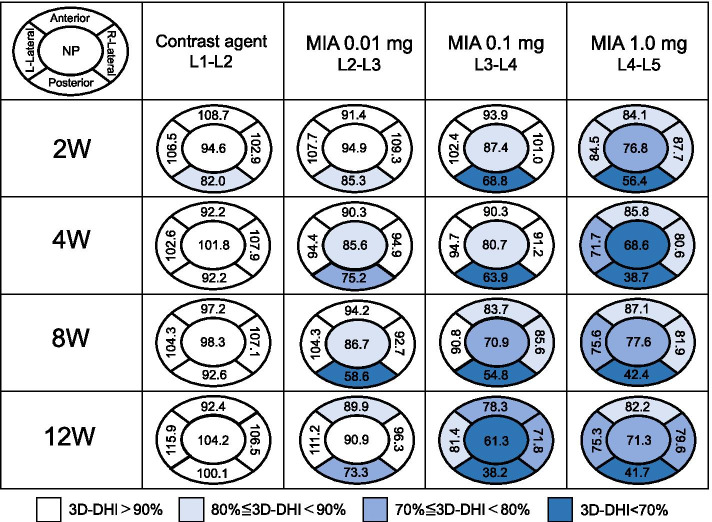
Change in three-dimensional (3D)-disc height index (DHI) of five anatomical zones. The percentage of the 3D-disc heights (DHs) of all five zones compared to those of L5–L6 discs was calculated as 3D-DHI: (3D-DH of each disc) / (3D-DH of L5–L6) × 100 (%). The 3D-DHI was evaluated in all the discs injected with contrast agent (CA) or monosodium iodoacetate (MIA) after 2, 4, 8, and 12 weeks post-injection. All five zones were divided into four groups depending on the degree of 3D-DHI as follows: 3D-DHI<70%, 70%≦3D-DHI<80%, 80%≦3D-DHI<90%, 90%≦3D-DHI

In the anatomical zone analysis, the 3D-DHI was calculated at each disc level and is shown as a color map (Fig. 5). In the CA control (L1–L2) discs, the 3D-DHI of all five zones at each timepoint was more than 90%, except for the posterior zone at week 2 (Fig. 5). In MIA 0.01 mg-injected discs (L2–L3), the area with 3D-DHI<90% was mainly identified at posterior and NP zones, but not at lateral (right and left) zones. In the MIA 0.1 mg-injected discs (L3–L4), the 3D-DHI of posterior zones time-dependently decreased from 2 weeks (68.8 %) to 12 weeks (38.2%) after injection. A time-dependent decrease of 3D-DHI from 87.4% at 2 weeks to 61.3% at 12 weeks was found at the NP zone. The area with 3D-DHI<90% was also identified at anterior and lateral zones 8 and 12 weeks after the injection. In the MIA 1.0 mg-injected discs (L4–L5), the 3D-DHI of all five zones was less than 90% from week 2 to week 12. Notably, the 3D-DHI of posterior zones decreased from 56.4% at week 2 to 41.7% at week 12.

### MRI assessment

**Fig. 6:**
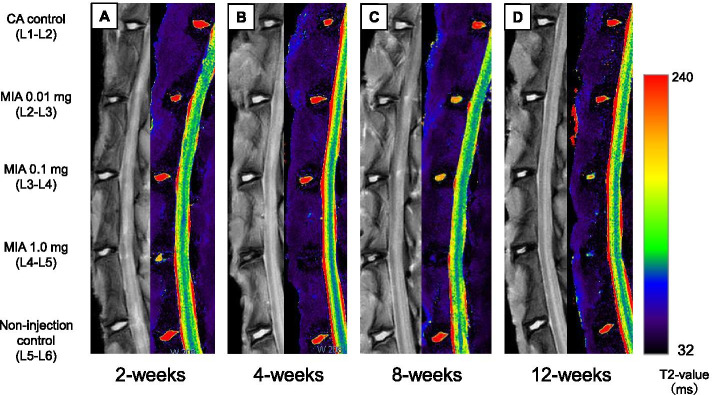
Representative sagittal magnetic resonance T2-weighted images (left panels) and T2 maps (right panels) of rabbit lumbar spines. **A** 2 weeks, **B** 4 weeks, **C** 8 weeks, and **D** 12 weeks after the injection of contrast agent (CA) and/or monosodium iodoacetate (MIA)

Representative sagittal images of color-coded T2 maps show a different degree of signal intensity of the NP, depending on disc level and timepoint (Fig. 6). Higher T2 values, as indicated by red pixels, were uniformly observed in the NP of CA (L1–L2) and NI control (L5–L6) discs throughout the experimental period. However, red pixels, replaced by orange or yellow pixels, were identified in the NP of MIA 0.01 mg- or 0.1 mg-injected discs at 8 and 12 weeks post-injection. A significant loss of red pixels, replaced by blue to black pixels, was found in the discs injected with 1.0 mg of MIA.

**Fig. 7:**
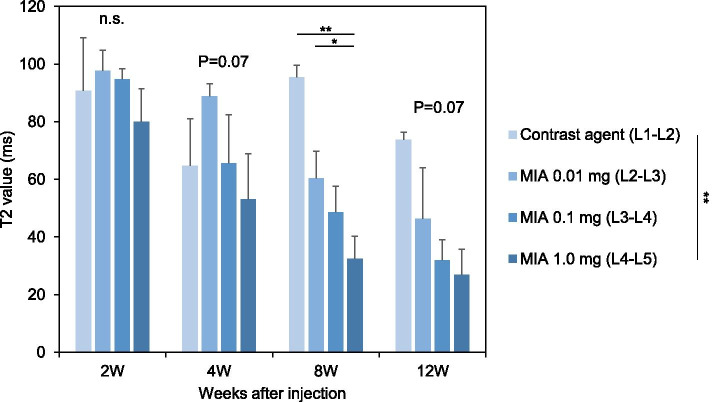
Changes in T2 values of rabbit intervertebral discs (IVDs). Data are expressed as mean ± standard error of the mean (SEM). Two-way analysis of variance and post hoc Bonferroni multiple comparisons test were used to compare all groups. *P < 0.05, **P < 0.01

Two-way repeated-measures ANOVA showed that the T2 value of the whole disc time-dependently decreased (*P* < 0.01) and differed significantly depending on the disc level during the observation period (*P* < 0.01) (Fig. 7). T2 values of CA control (L1–L2) discs showed no significant changes compared to NI controls (L5–L6) discs. The intradiscal injection of MIA 1.0 mg (L4–L5 discs) significantly decreased T2 values compared to those of CA control (L1–L2) discs (*P* < 0.01) (Fig. 7). Timepoint analysis showed significant differences in T2 values among the groups at week 8 post-injection (*P* < 0.01). The mean T2 value of MIA 1.0 mg-injected discs (L4–L5) was significantly lower than that of MIA 0.01 mg-injected discs (L2–L3) and CA control discs (MIA 0.01: *P* < 0.05, CA: *P* < 0.01) (Fig. 7). A similar trend of T2 values of MIA-injected discs was identified at 4 and 12 weeks post-injection; however, it did not reach statistical significance among groups (4 weeks: *P* = 0.07; 12 weeks: *P* = 0.07).

### Histological assessment

**Fig. 8:**
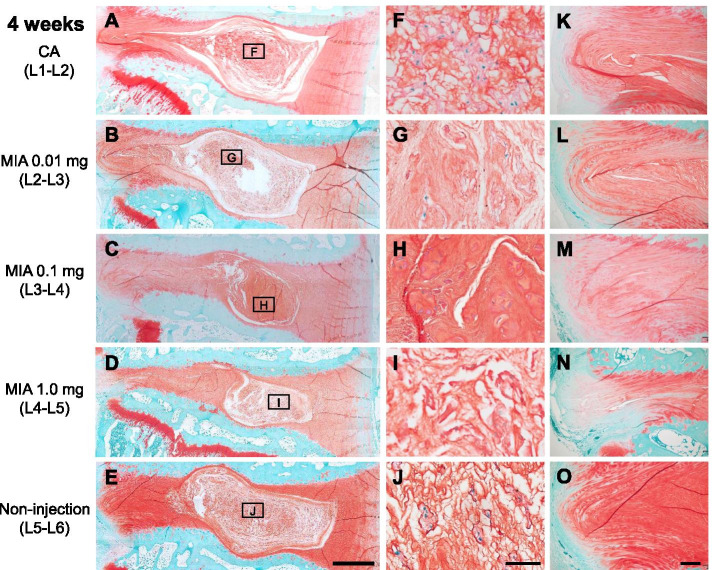
Representative histology of rabbit intervertebral discs 4 weeks post-injection of contrast agent (CA) (**A**, **F**, **K**) and/or monosodium iodoacetate (MIA) (**B–D**, **G–I**, **L–M**) stained with Safranin-O. L2–L3 discs (**B**, **C**, **D**) were injected with 0.01 mg of MIA, L3–L4 discs (**C**, **H**, **M**) were injected with 0.1 mg of MIA and L4–L5 discs (**D**–**N**) were injected with 1.0 mg of MIA. L5–L6 discs (**E**, **J**, **O**) were used for non-injection (NI) controls. **F–J** are high-magnification images of the nucleus pulposus (NP) indicated by a square. **K–O** are high-magnification images of the posterior annulus fibrosus (AF). Scale bars: 1 mm (**A–E**), 5 μm (**F–J**), 200 μm (**K–O**)

**Fig. 9:**
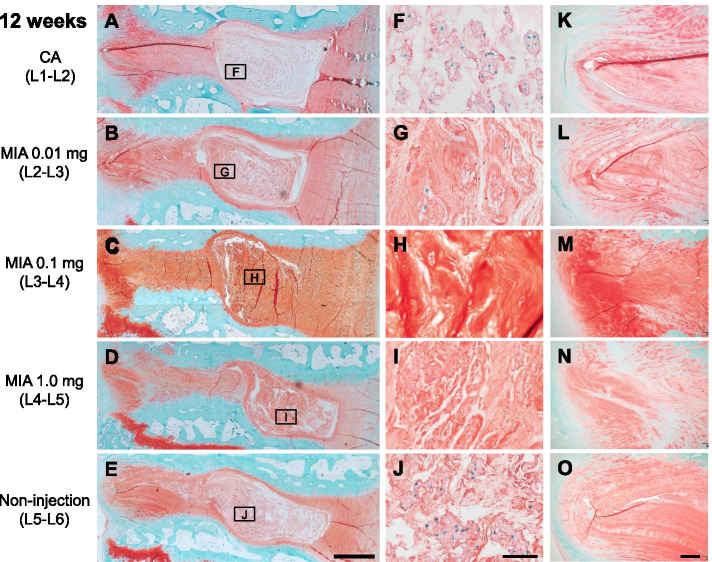
Representative histology of rabbit intervertebral discs 12 weeks post-injection of contrast agent (CA) (**A**, **F**, **K**) and/or monosodium iodoacetate (MIA) (**B–D**, **G–I**, **L–M**) stained with Safranin-O. L2–L3 discs (**B**, **C**, **D**) were injected with 0.01 mg of MIA, L3–L4 discs (**C**, **H**, **M**) were injected with 0.1 mg of MIA and L4–L5 discs (**D–N**) were injected with 1.0 mg of MIA. L5–L6 discs (**E**, **J**, **O**) were used for non-injection (NI) controls. **F–J** are high-magnification images of the nucleus pulposus (NP) indicated by a square. **K–O** are high-magnification images of the posterior annulus fibrosus (AF). Scale bars: 1 mm (**A–E**), 5 μm (**F–J**), 200 μm (**K–O**)

Representative histology with Safranin-O staining at 4 and 12 weeks post-injection is shown in Figs. 8 and 9. At 4 weeks post-injection, the CA (Fig. 8A) and NI control (Fig. 8E) discs showed a normal histological appearance of rabbit IVDs, displaying an intact AF with a regular pattern of fibrocartilage lamellas with a well-defined border between the AF and NP (Fig. 8K, O). The NP consisted of numerous NP (vacuolated) cells within a fine cotton-like ECM (Fig. 8F, J). The intradiscal injection of MIA induced significant changes in histological appearance, especially in the NP at 4 weeks post-injection. MIA 0.01 mg or 0.1 mg induced a slight to a moderate decrease of NP cells with slight condensation of the ECM (Fig. 8G, H). MIA 1.0 mg induced a severe decrease of NP cells with moderate condensation of the ECM (Fig. 8I). MIA 1.0 mg also affected the histology of the posterior AF with partial ruptures and a serpentine pattern of fibers (Fig. 8N); this was not found in the anterior AF.

At 12 weeks post-injection, the progression in histological changes induced by MIA was observed in both NP and AF tissues (Fig. 9). MIA 0.01 mg induced a moderate decrease of NP cells with local condensation of the ECM (Fig. 9G); AF fibers in posterior AF tissues were partially ruptured (Fig. 9L). MIA 0.1 mg and 1.0 mg induced a collapse of the NP tissue, with a significant decrease of NP cells and severe condensation of the ECM (Fig. 9H, I). Wavy fibrocartilage lamellas of the posterior AF with severely ruptured fibers were observed (Fig. 9M, N).

**Fig. 10:**
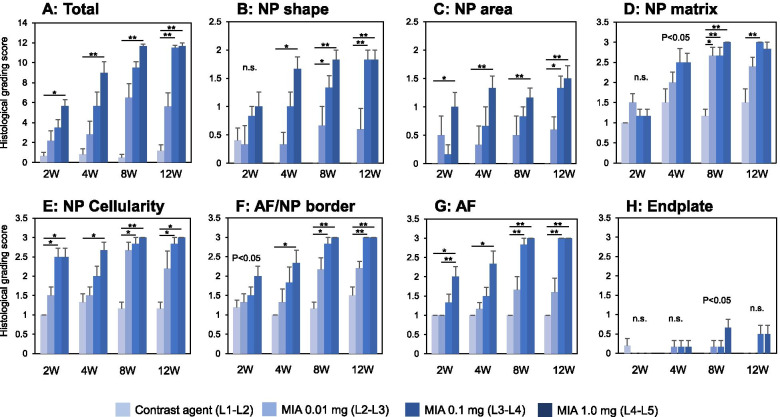
Histological grading scores. In rabbit intervertebral discs (IVDs) injected with contrast agent (CA) and/or monosodium iodoacetate (MIA) at 2, 4 , 8 , and 12 weeks post-injection, histological grading scores were evaluated using the standardized histopathology scoring system of rabbit IVD degeneration [26–28] for the total of seven categories: **A** total of seven categories, **B** nucleus pulposus (NP) shape, **C** NP area, **D** NP matrix, **E** NP cellularity, **F** border between the annulus fibrosus (AF) and NP, **G** AF, and **H** endplate. Data are expressed as mean ± standard error of the mean (SEM). The Kruskal-Wallis test was used to compare all groups. *P < 0.05, **P < 0.01

The intradiscal injection of MIA significantly affected the total histological score of rabbit disc degeneration [27] throughout the experimental period (weeks 2, 4, 8, and 12: all *P* < 0.01) (Fig. 10A). There were no significant differences on total histological scores between the CA and NI controls.

The total histological scores of MIA 0.1 mg-injected discs were significantly higher than those in the CA control discs at week 12 (*P* < 0.01) (Fig. 10A). The intradiscal injection of MIA 1.0 mg significantly increased the total histological scores compared to those of the CA control discs (week 2: *P* < 0.05, week 4 to 12: *P* < 0.01, Fig. 10A). Histological grading scores of each of the seven categories, including the NP shape (Fig. 10B), NP area (Fig. 10C), NP matrix (Fig. 10D), NP cellularity (Fig. 10E), AF/NP border (Fig. 10F), AF (Fig. 10G), and endplate (EP) (Fig. 10H), are separately shown in Fig. 10.

### Cellular changes in nucleus pulpous cells

**Fig. 11:**
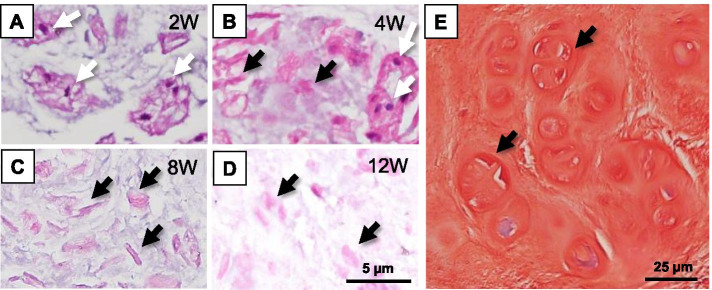
Representative histology of nucleus pulposus cells. Histology of cellular changes in the nucleus pulpous (NP) of MIA 1.0 mg-injected discs after 2 (**A**), 4 (**B**), 8 (**C**), and 12 weeks (**D**) post-injection stained with hematoxylin-eosin are representatively shown. Scale bar: 5 μm (**A–D**). Safranin-O staining histology of cell clones in the NP of MIA 0.01 mg-injected disc is representatively shown (**E**). Scale bar: 25 μm (**E**)

Cellular changes in the NP cells of MIA 1.0 mg-injected discs are representatively shown in Fig. 11(A-D). Many NP cells with strong hematoxylin-stained nuclei in the eosin-stained cytoplasm were found 2 weeks post-injection (Fig. 11A). However, at 4 weeks post-injection, a substantial number of NP cells with weak hematoxylin-stained nuclei (Fig. 11B), flattening of nuclei (Fig. 11C), and disintegration of nuclei (Fig. 11D) were detected.

At 12 weeks post-injection, the number of surviving cells in the MIA 0.01 mg-injected discs decreased to 40.6% of those in CA control discs, that of MIA 0.1 mg-injected discs was decreased to 19.1 % of those in CA control discs, and that of MIA 1.0 mg-injected discs was decreased to 2.5% of those in CA control discs.

On the other hand, cell clones in NP tissues (Fig. 11E) were observed mainly in MIA 0.01 mg-injected discs (L2–L3) and MIA 0.1 mg-injected discs (L3–L4). In MIA 0.01 mg-injected discs, cluster formation was observed in 33.3% (2/6) of discs at 2 and 4 weeks, and 50% (3/6) of discs at 12 weeks. Cell clusters in the NP were also found in 33.3% (2/6) of MIA 0.1 mg-injected discs at 4 weeks post-injection and 33.3% (2/6) at 12 weeks post-injection. No cell clones were detected in discs injected with MIA 1.0 mg.

### Correlation between MRI T2 values and histological score

MRI T2 values negatively correlated with the total histological score (*r* = −0.83, *P* < 0.01). The subclass analysis showed that MRI T2 values significantly correlated with NP shape (*r* = −0.66, *P* < 0.01), NP area (*r* = −0.75, *P* < 0.01), NP matrix (*r* = −0.81, *P* < 0.01), NP cellularity (*r* = −0.67, *P* < 0.01), AF/NP border (*r* = −0.78, *P* < 0.01), AF (*r* = −0.80, *P* < 0.01), and EP (*r* = −0.32, *P* < 0.01).

## Discussion

For the first time, we have evaluated radiological and histological changes of rabbit IVDs after an intradiscal injection of MIA. This study showed that disc height, especially that of the posterior AF zone, time- and dose-dependently decreased, and MRI analysis showed a tendency for decreased T2 values in a time- and dose-dependent manner. Our results also showed that histological grading scores exacerbated time- and dose-dependently after the injection of MIA. Importantly, we found that MIA induced a high percentage of cell death in the NP region.

Clinically, disc height narrowing on lumbar radiographs is a standard indicator for IVD degeneration [12]. To evaluate disc height, the ratio of disc height to that of the vertebral body, defined as “disc height index,” has been used [22]. In this study, the actual value of disc height was directly measured by calibrating with a 10-mm metal wire. Our results showed that no significant differences in disc height change against baseline, as represented by “percent disc height (%DH),” between the CA control (L1–L2) and NI control (L5–L6) discs were found throughout the experimental period. This suggests that the intradiscal injection of CA using a 31-gauge needle does not influence disc height change in this animal model.

The results of the radiographic evaluation showed that the intradiscal injection of MIA induced a progressive and temporal decrease of disc height. When comparing the %DH among three different concentrations of MIA-injected discs, the intradiscal injection of MIA 0.01 mg mildly decreased disc height compared to that of MIA 0.1 mg- and 1.0 mg-injected discs, finally decreasing to approximately 80% of baseline. On the other hand, the two higher doses of MIA (0.1 and 1.0 mg) induced a final decrease in disc height to approximately 50% of baseline; however, no significant difference was found between the two doses. These results suggest that there would be significant differences in the effect of disc height narrowing between the administration of low-dose MIA (0.01 mg) and high dose MIA (0.1 and 1.0 mg) into the rabbit IVDs, with a similar effect between MIA 0.1 and 1.0 mg. Accordingly, MIA 0.1 mg would be a necessary and sufficient dose for the severe disc height narrowing of rabbit IVDs.

Due to the three-dimensional nature of the intervertebral structure, disc height varies depending on the area of measurement [23]. 3D quantification of disc height would be ideal and more accurate than measurement by radiography [30]. Therefore, the 3D-DHI of whole and five anatomical zones was evaluated by 3D-CT analysis. The change in 3D-DHI following MIA injection showed a tendency similar to that of the %DHI evaluated by lumbar radiography. The results of the anatomical zone analysis showed that the intradiscal injection of MIA predominantly induced disc height narrowing at the posterior AF zone. The low-dose MIA (0.01mg) mainly decreased the posterior AF zone; however, a higher dose of MIA (0.1 and 1.0 mg) affected not only the posterior zone but also the NP, lateral, and anterior zones. Tsuji et al. [31] conducted a microscopic observation of AF tissues of young human IVDs and showed that the number of lamellar bundles in the anterior AF was higher than that in the posterior AF. Furthermore, a high percentage of incomplete and/or discontinuous lamellar bundles was found in the posterior AF tissues, suggesting the mechanical friability of posterior human AF tissue. Similar morphological and histological characteristics were also identified in rabbit AF tissues. Therefore, the authors speculated that MIA would induce a structural failure primarily in the posterior AF tissues, which leads to non-uniform disc height narrowing depending on the disc zones.

MRI T2-mapping has been shown to quantitatively evaluate changes in water and proteoglycan (PG) content and arrangement of the collagen network structure of human IVDs [32, 33]. Notably, previous studies have shown that T2 values in human IVDs have a significant correlation with the degree of disc degeneration [10, 33, 34]. Our results of MRI T2 mapping showed that the intradiscal injection of MIA significantly affected changes in MRI T2 values of rabbit IVDs. MIA induced a dose-dependent decrease in MRI T2-values at 4, 8, and 12 weeks post-injection, suggesting that MIA decreases water content and induces changes in the molecular composition of the extracellular matrix (ECM) of rabbit IVDs.

Two weeks post-injection of MIA-injected discs, histological analyses showed that the ECM in the NP lesion showed only a mild change while maintaining a fine cotton-like appearance. However, the condensational change of the ECM progressed time-dependently until 12 weeks post-injection in all doses of MIA-injected discs. These time- and dose-dependent changes of histology in the NP would be responsible for the changes in MRI T2 values. Our results also showed that MRI T2 values were strongly correlated with total histological grading scores and subclass grading scores, except for the “EP.” Takahashi et al. [35] injected MIA (0.2 mg and 1.0 mg) into rat knee joints and evaluated the histopathological changes in articular cartilage. They reported that substantial numbers of chondrocyte death, identified by abnormality of nuclei, were detected in both doses of MIA-injected knees and that 0.2 mg of MIA induced a mild osteoarthritis change while 1.0 mg of MIA induced end-stage osteoarthritic changes. Histological changes of rat articular cartilage after the injection of MIA shared features common to the histological results of our rabbit disc study.

Partial ruptures and serpentine patterns of fibers were mainly found in posterior AF tissues of MIA 0.1 mg- and 1.0 mg-injected discs, while no remarkable histological changes were identified in anterior AF tissues. These histological findings would be attributed to the decreased disc height at the posterior AF zone as shown by 3D-DH analysis. Boos et al. [36] semi-quantitatively evaluated the age-related histological changes of human IVD tissues and reported that degenerative changes that started in the second decade of life were initially found in the NP. Cell death and chondrocyte proliferation were found in samples from children and temporally increased to adulthood. The substantial increase in cell death associated with extensive chondrocyte proliferation was found in IVDs from teenagers and was most pronounced in samples from adults 30 to 50 years old [36]. In our rabbit study, cell cloning (cluster formation) was locally detected in approximately 30–50% of the NP of discs injected with MIA (0.01 and 0.1 mg), but not with MIA (1.0 mg). Hence, we recognized that the cell proliferative activity of the rabbit NP would be impaired after MIA injection; this would be different from the histopathologic changes found in human IVD degeneration.

Jiang et al. [17] examined the cytotoxic effect of MIA on rat chondrocytes in vitro and showed that MIA treatment induced apoptosis in chondrocytes by upregulating cytochrome *c* and caspase-3 protein levels. The results of these previous reports and our study suggest that intradiscal injection of MIA into rabbit IVDs would induce degenerative tissue changes caused by cell death in rabbit IVDs.

Suh et al. [21] reported that the intradiscal injection of MIA increased nuclear factor-kappa B (NF-κB) and cyclooxygenase-2 (COX-2) expression in rat IVDs and dorsal root ganglia (DRGs); these are the chief regulators of catabolic factors and inflammatory pain. Therefore, we speculated that the administration of MIA might also have a catabolic effect on rabbit IVD cells and induce discogenic pain; this would be worth investigating in a future study.

Because cell death is one of the characteristics of this MIA-induced IVD degeneration model, there is the possibility that this model might be appropriate for evaluating cell replacement therapies, such as the use of mesenchymal stem cells [37] or progenitor cells [38]. While administering a high dose of MIA (0.1 or 1.0 mg) resulted in an advanced stage of IVD degeneration, this could be utilized to test tissue-engineered IVDs or intervertebral implants.

There are several limitations to this study. First, we have evaluated the effect of MIA on rabbit IVD degeneration until 12 weeks; however, the long-term effects of MIA have not been evaluated. Second, it has been reported that the response of rabbit IVDs against external stimuli, such as disc puncture, differs among disc levels, probably due to differences in disc volume [39]. Therefore, the effect of MIA on rabbit IVDs might differ among L2–L3, L3–L4, and L4–L5 discs.

## Conclusions

For the first time, we have shown that the intradiscal injection of MIA induced degenerative changes of rabbit IVDs evaluated by radiography, CT, MRI, and histology. The progression of disc height narrowing that was evaluated by radiography and CT and the decrease in MRI T2 values induced by MIA injection would be like those of human IVD degeneration. However, MIA induced cell death in the rabbit NP with a high percentage, while cell proliferation (cell cloning) was low; these cellular changes would differ from the pathophysiology of human IVD degeneration. Nevertheless, based on the dose-dependent degenerative changes induced by MIA, MIA injection into rabbit IVDs could be used as an animal model of IVD degeneration.

## Data Availability

The datasets used and/or analyzed during the current study are available from the corresponding author on reasonable request.

## Abbreviations

AF: Annulus fibrosus
3D-DH: Three-dimensional disc height
ECM: Extracellular matrix
IVD: Intervertebral disc
MAPK: Mitogen-activated protein kinase
MIA: Monosodium iodoacetate
μCT: Micro-computed tomography
MRI: Magnetic resonance imaging
NP: Nucleus pulposus
PG: Proteoglycan
